# Apoptosis Induction of Human Prostate Carcinoma DU145 Cells by Diallyl Disulfide *via* Modulation of JNK and PI3K/AKT Signaling Pathways

**DOI:** 10.3390/ijms131114158

**Published:** 2012-11-02

**Authors:** Dong Yeok Shin, Gi-Young Kim, Jun Hyuk Lee, Byung Tae Choi, Young Hyun Yoo, Yung Hyun Choi

**Affiliations:** 1 Dongnam Institute of Radiological & Medicine Sciences, Busan 619-953, Korea; E-Mail: bboglyang@hanmail.net; 2 Laboratory of Immunobiology, Department of Marine Life Sciences, Jeju National University, Jeju 690-756, Korea; E-Mail: immunkim@cheju.ac.kr; 3 Biotechnology Examination Division, Chemistry and Biotechnology Examination Bureau, Korean Intellectual Property Office, Daejeon 302-701, Korea; E-Mail: junhlee@kipo.go.kr; 4 Division of Meridian and Structural Medicine, School of Korean Medicine, Pusan National University, Yangsan 626-870, Korea; E-Mail: choibt@pusan.ac.kr; 5 Department of Anatomy and Cell Biology and Mitochondria Hub Regulation Center, College of Medicine, Dong-A University, Busan 602-714, Korea; 6 Department of Biochemistry, College of Oriental Medicine and Anti-Aging Research Center, Dongeui University, Busan 614-052, Korea

**Keywords:** diallyl disulfide, apoptosis, MAPK, PI3K/Akt

## Abstract

Diallyl disulfide (DADS), a sulfur compound derived from garlic, has various biological properties, such as anticancer, antiangiogenic and anti-inflammatory effects. However, the mechanisms of action underlying the compound’s anticancer activity have not been fully elucidated. In this study, the apoptotic effects of DADS were investigated in DU145 human prostate carcinoma cells. Our results showed that DADS markedly inhibited the growth of the DU145 cells by induction of apoptosis. Apoptosis was accompanied by modulation of Bcl-2 and inhibitor of apoptosis protein (IAP) family proteins, depolarization of the mitochondrial membrane potential (MMP, *ΔΨm*) and proteolytic activation of caspases. We also found that the expression of death-receptor 4 (DR4) and Fas ligand (FasL) proteins was increased and that the level of intact Bid proteins was down-regulated by DADS. Moreover, treatment with DADS induced phosphorylation of mitogen-activated protein kinases (MAPKs), including extracellular-signal regulating kinase (ERK), p38 MAPK and c-Jun *N*-terminal kinase (JNK). A specific JNK inhibitor, SP600125, significantly blocked DADS-induced-apoptosis, whereas inhibitors of the ERK (PD98059) and p38 MAPK (SB203580) had no effect. The induction of apoptosis was also accompanied by inactivation of phosphatidylinositol 3-kinase (PI3K)/Akt and the PI3K inhibitor LY29004 significantly increased DADS-induced cell death. These findings provide evidence demonstrating that the proapoptotic effect of DADS is mediated through the activation of JNK and the inhibition of the PI3K/Akt signaling pathway in DU145 cells.

## 1. Introduction

Garlic, *Allium sativum*, is a common plant used mainly as food and has recently been reported to have medicinal attributes, including antihypertensive, antiatherosclerotic and antioxidant properties [1–5]. Epidemiological studies and laboratory experiments have recently demonstrated that sulfur-containing compounds, such as S-allyl cysteine, diallyl sulfide and diallyl disulfide (DADS), which contains two sulfur atoms and diallyl trisulfide, all of which are major components of garlic, may be associated with a reduced risk of certain cancers [6–9]. Among these, the biological activity of DADS, including its anticancer and anti-inflammatory effects, has been shown to be stronger [10–14]. In particular, this compound is known to inhibit the proliferation of various types of human cancer cells, through the induction of cell cycle arrest or apoptosis [15–22].

Apoptosis, or programmed cell death, is a highly regulated process that allows a cell to self-degrade to enable the body to eliminate unwanted or dysfunctional cells. Therefore, elucidation of the apoptotic mechanism is important for the prevention and cure of various, currently incurable diseases, including cancer [23–25]. Moreover, apoptosis suppression in various cancer cells is known to cause tumor growth and resistance to cytotoxic anticancer agents. Two different apoptotic pathways have been described to date: the death-receptor (extrinsic) pathway and the mitochondrial (intrinsic) pathway [26–28]. The extrinsic pathway is initiated through the stimulation of transmembrane death receptors located on the cell membrane. In contrast, the intrinsic pathway is initiated through the release of signal factors by mitochondria within the cell, and its two apoptotic pathways are executed mainly by a class of cysteine proteases known as caspases [29–31]. According to recent studies, many chemopreventive and/or chemotherapeutic agents can cause cell death via the induction of apoptosis. Therefore, the induction of apoptotic cell death is an important mechanism in the anticancer properties of many drugs.

Although the induction of apoptosis by DADS has been observed in some cancer cell lines, the molecular mechanisms of DADS’s proapoptotic action are not fully understood. In the present study, we attempted to elucidate the proapoptotic potential of DADS and underlying intracellular signal transduction pathways involved in inducing the apoptosis of DU145 human prostate cancer cells. Our results demonstrated that DADS, together with the death receptor-mediated extrinsic pathway, triggers the apoptosis of DU145 cells through the activation of the intrinsic caspase pathway, accompanied by the activation of the c-Jun *N*-terminal kinase (JNK) and the suppression of the phosphatidylinositol 3-kinase (PI3K)/Akt pathway.

## 2. Results and Discussion

### 2.1. Inhibition of Cell Viability by DADS in DU145 Cells

**Figure 1:**
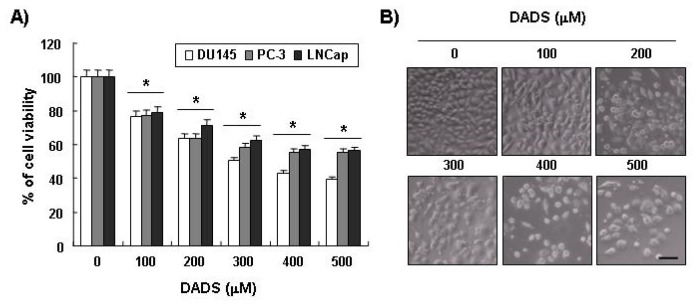
Effects of DADS on cell viability in human prostate cancer cell lines. (**A**) The cells (DU145, PC-3 and LNCap) were treated with the indicated concentrations of DADS for 48 h. The cell viability was measured by the metabolic-dye-based MTT assay. Each point represents the mean ± SD of three independent experiments. The significance was determined by the Student’s *t*-test (*p < 0.05 *vs.* untreated control). (**B**) DU145 cells grown under the same conditions as (**A**) were sampled and photographed under an inverted microscope (original magnification 200×). Scale bar is 50 μM.

To examine the effects of DADS on the proliferation of human prostate carcinoma cells, three types of prostate carcinoma cells (DU145, PC3 and LNCap) were treated with appropriate concentrations of DADS for 48 h and then subjected to MTT assays. As shown in the results shown in Figure 1A, as the concentration of DADS treatment increased, cell viability decreased in all the three types of cells. For instance, when DU145 cells were treated with 200 μM and 400 μM of DADS, their cell viability decreased by around 35% and 60%, respectively, compared to the control group. In particular, since DU145 cells showed higher sensitivity to DADS compared to PC3 and LNCap cells, later experiments were conducted with DU145 cells.

### 2.2. Induction of Apoptosis by DADS in DU145 Cells

**Figure 2:**
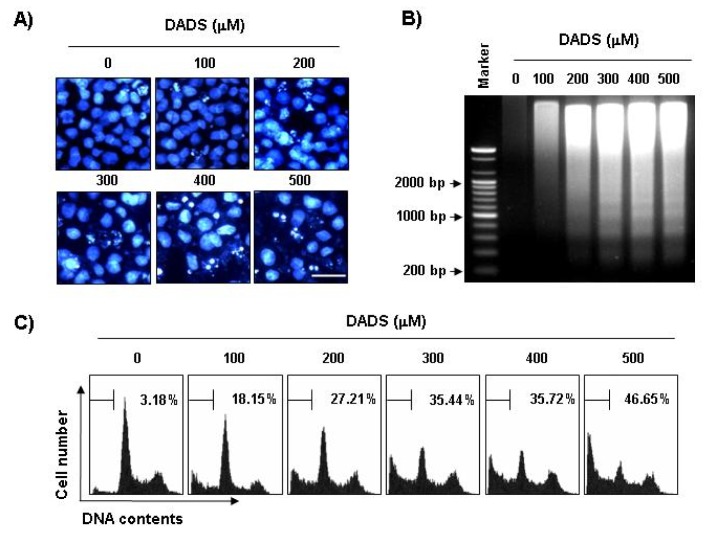
Induction of apoptosis by DADS in DU145 cells. (**A**) The cells were incubated with the indicated concentrations of DADS for 48 h, fixed and stained with DAPI solution. The stained nuclei were observed under a fluorescent microscope (original magnification, 400×). Scale bar is 50 μM; (**B**) For the analysis of DNA fragmentation, genomic DNA from cells grown under the same conditions as (**A**) was extracted, separated by 1.0% agarose gel electrophoresis and visualized under UV light after staining with ethidium bromide (EtBr). The DNA marker indicates the size of the fragments of the DNA ladder. The results shown are from one representative experiment of two experiments that showed similar patterns; (**C**) To quantify the degree of apoptosis induced by DADS, cells were evaluated by flow cytometry for sub-G1 DNA content, which represents the cells undergoing apoptotic DNA degradation. The data are the mean ± SD of the two different experiments.

Next, we performed experiments to determine whether this inhibitory effect of DADS on DU145’s cell growth resulted from apoptotic cell death. To examine apoptosis morphologically, we stained the nuclei of untreated and DADS-treated cells with 4,6-Diamidino-2-phenyllindile (DAPI) solution. As shown in Figure 2A, the cells treated with DADS exhibited significant chromatin condensation, loss of nuclear construction and formation of apoptotic bodies in a concentration-dependent fashion, whereas we did not observe these features in control cells. Second, we analyzed DNA fragmentation, which is another hallmark of apoptosis. Following agarose gel electrophoresis of DNA from cells treated with DADS, we observed a typical ladder pattern of internucleosomal fragmentation. In contrast, there was barely any evidence of DNA fragmentation in the control cells (Figure 2B). In addition, to detect hypodiploid cell populations, we conducted flow-cytometric analysis to determine the degrees of apoptosis in the cells treated with DADS. As indicated in Figure 2C, the addition of DADS to the DU145 cells resulted in increased accumulation of cells in the sub-G1 phase in a manner similar to that observed with DADS, inhibiting the cell’s viability and leading to the formation of apoptotic bodies and the accumulation of extranuclear fragmented DNA. This finding suggests that DU145 cells may undergo apoptosis after exposure to DADS and that there is a good correlation between the extent of apoptosis and the inhibition of growth.

### 2.3. Effects of DADS on the Expression of Apoptosis-Related Proteins in DU145 Cells

**Figure 3:**
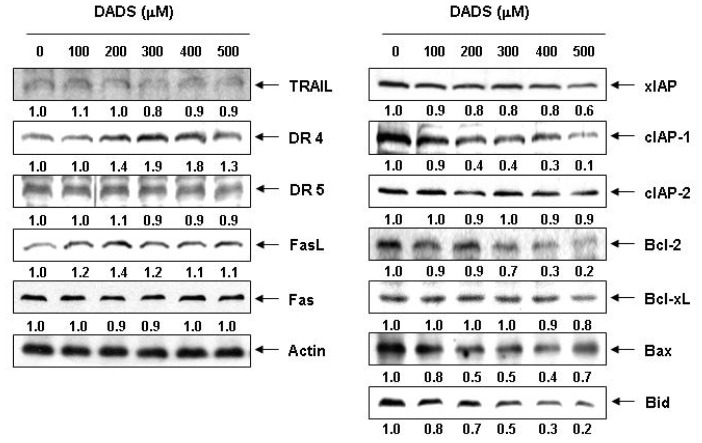
Effect of DADS on the levels of death receptor-related, Bcl-2 and IAP family proteins in DU145 cells. Cells were incubated with the indicated concentrations of DADS for 48 h. The cells were lysed, and the cellular proteins were then separated by SDS-polyacrylamide gels and transferred onto nitrocellulose membranes. The membranes were probed with the indicated antibodies. The proteins were visualized using an ECL detection system. Actin was used as an internal control. The numbers represent the average densitometric analyses as compared with actin in, at a minimum, two or three different experiments.

To identify the pathway involved in the apoptosis of the DADS-treated DU145 cells, we measured the protein expression of the death receptor-related, the Bcl-2 and the IAP family of proteins by Western blotting. As shown in Figure 3, the results showed that DADS treatment resulted in a concentration-dependent increase in the levels of DR4 and FasL. Under the same conditions, DADS treatment markedly inhibited the levels of the antiapoptotic IAP family of proteins such as XIAP and cIAP-1, both of which bind to caspases and lead to their inactivation, in a concentration-dependent manner. Among the Bcl-2 family proteins, the levels of antiapoptotic Bcl-2 proteins, which function as critical proteins to maintain the stabilization of mitochondria, markedly decreased in response to DADS treatment. In addition, although we did not detect the truncated form of the proapoptotic protein Bid, a BH3-only protein, DADS decreased the whole form of Bid proteins, reflecting Bid cleavage and activation (Figure 3).

### 2.4. Decrease in the Levels of MMP by DADS in DU145 Cells

**Figure 4:**
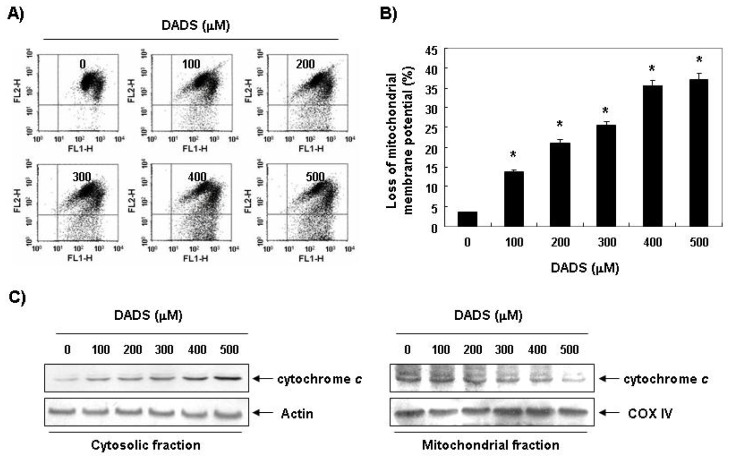
Effects of DADS on the levels of MMP in DU145 cells. (**A**) The cells were treated with the indicated concentrations of DADS for 48 h. They were collected and incubated with JC-1 (10 μM) for 20 min at 37 °C in the dark. The cells were then washed once with PBS and analyzed by a DNA flow cytometer; (**B**) The results are presented as the mean ± SD of three independent experiments. The significance was determined by the Student’s *t*-test (*p < 0.05 *vs.* untreated control); (**C**) The cytosolic and mitochondrial proteins were extracted cells grown under the same conditions and analyzed by Western blotting using the indicated antibodies. Actin and COX4 were used as internal controls for the cytosolic and mitochondrial fractions, respectively.

Mitochondria, which play an essential role in apoptosis, are specialized organelles. They contain an outer membrane separated from an inner membrane by an intermembrane space, which contains many proapoptotic proteins, including cytochrome *c*. A decrease in the levels of MMP causes disruption of the outer mitochondrial membrane, which, in turn, contributes to the release of cytochrome *c*. Therefore, we next examined the levels of MMP as well as the levels of cytosolic and mitochondrial cytochrome *c* to characterize the role of mitochondria in DADS-induced apoptosis. As shown in Figure 4, MMP showed a concentration-dependent decrease by DADS treatment. In addition, exposure of DU145 cells to DADS led to a significant increase in the level of cytosolic release of the mitochondrial pro-apoptotic protein cytochrome *c*, indicating that DADS induced mitochondrial membrane hyperpolarization by depolarization.

### 2.5. Activation of Caspases by DADS in DU145 Cells

**Figure 5:**
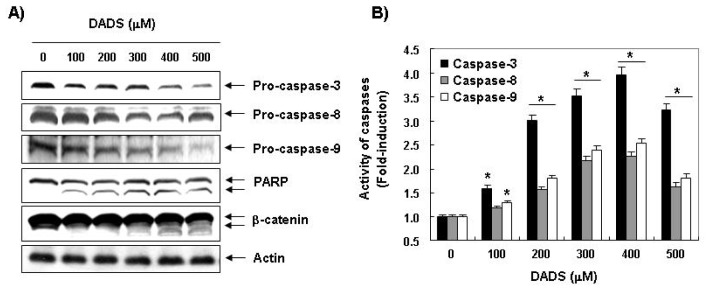
Activation of caspases and the degradation of PARP and β-catenin by DADS in DU145 cells. (**A**) Cells were treated with the indicated concentrations of DADS for 48 h. The cells were lysed, and the cellular proteins were visualized using the indicated antibodies and an ECL detection system. Actin was used as an internal control; (**B**) After 48 h incubation with the indicated concentrations of DADS, the cells were lysed, and aliquots (50 μg protein) were assayed for *in vitro* caspase-3, -8 and -9 activity using DEVD-pNA, IETD-pNA and LEHD-pNA as substrates, respectively, at 37 °C for 1 h. The released fluorescent products were measured. The data are expressed as the mean ± SD of three independent experiments. The significance was determined by the Student’s *t*-test (*p < 0.05 *vs.* untreated control).

We measured the expression levels and the activities of caspase-3, -8 and -9 in the DU145 cells exposed to DADS to determine whether DADS-induced apoptosis is associated with the activation of the caspases. As shown in Figure 5A, immunoblotting results showed that DADS treatment induced a concentration-dependent decrease in the levels of the procaspase-3, -8 and -9 proteins. For further quantification of the proteolytic activation of caspases, proteins in the lysates of cells treated with DADS were normalized and then assayed for *in vitro* activities using fluorogenic substrates. The data indicated that treatment with DADS resulted in a significant concentration-dependent increase in the activities of caspase-3, -8 and -9 compared with the control cells (Figure 5A). Furthermore, subsequent Western blot analysis revealed that progressive proteolytic cleavage products of PARP and β-catenin, downstream target proteins of the activated caspase-3 occurred in the DU145 cells treated with DADS (Figure 5A), demonstrating an association of DADS-induced apoptosis with caspase activation.

### 2.6. Activation of the JNK Pathway in DADS-Induced Apoptosis of DU145 Cells

**Figure 6:**
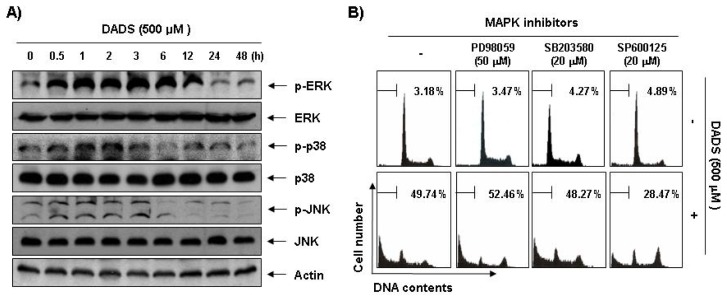
Effects of the activation of MAPKs on DADS-induced apoptosis in DU145 cells. (**A**) The cells were treated with DADS (500 μM) for the indicated times. They were then lysed, and equal amounts of cell lysates were resolved by SDS-polyacrylamide gels, transferred to nitrocellulose and probed with the indicated antibodies; (**B**) The cells were pretreated with the indicated MAPK inhibitors (SB203580, 20 μM; SP600125, 20 μM; and PD98059, 50 μM) for 1 h and then treated with DADS (500 μM) for 48 h. The percentage of sub-G1 population was evaluated by a flow cytometer. The data are the mean ± SD of two different experiments.

Next, we investigated the effect of DADS treatment on the expression and activities of MAPKs to determine whether these signaling pathways play a role in mediating the observed apoptotic response. As Figure 6A demonstrates, stimulation of the DU145 cells with DADS led to rapid phosphorylation of ERK, p38 MAPK and JNK, with peak levels of each phospho-MAPK observed 1 to 3 h after the addition of DADS. To confirm the association between the activation of MAPKs and apoptosis induction by DADS, we pretreated the cells with MAPK inhibitors and analyzed the sub-G1 DNA content by a flow cytometer. As shown in Figure 6B, pretreatment with SP600125 (a potent inhibitor of JNK) significantly reduced the increased number of cells with sub-G1 DNA content by DADS. However, pretreatment with PD98059 (a potent inhibitor of ERK) or SB203589 (a specific inhibitor of the p38 MAPK) did not have a significant effect on DADS treatment.

### 2.7. Inactivation of the PI3K/Akt Pathway in DADS-Induced Apoptosis of DU145 Cells

**Figure 7:**
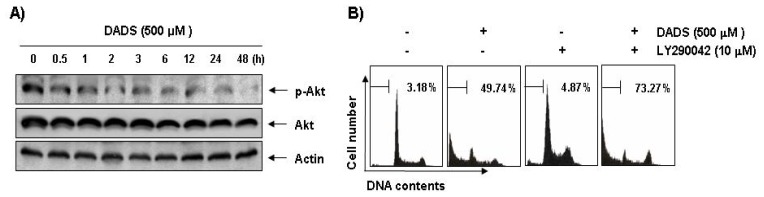
DADS triggers apoptosis through PI3K/Akt signaling in DU145 cells. (**A**) The cells were treated with DADS (500 μM) for the indicated times. Equal amounts of cell lysate were resolved by SDS-polyacrylamide gels, transferred to nitrocellulose membranes and probed with the anti-p-Akt and anti-Akt antibodies. The proteins were visualized using an ECL detection system; (**B**) The cells were stimulated with 500 μM DADS for 48 h after pretreatment of 10 μM LY290042 for 1 h. The degree of apoptosis was determined by a flow cytometer. Each point represents the mean ± SD of two independent experiments.

To investigate the possible involvement of the PI3K/Akt pathway in DADS-induced apoptosis, we assessed phosphorylated Akt levels during DADS-induced apoptosis in the DU145 cells. Western blot analysis showed that Akt was constitutively active in the DU145 cells and that phosphorylated Akt levels were significantly decreased in response to DADS treatment. However, there were no effects on steady-state levels of total Akt protein in the DU145 cells treated with DADS (Figure 7A). To confirm the involvement of the PI3K/Akt pathway in DADS-induced apoptosis, we investigated whether DADS significantly induces apoptosis in the presence of LY290042, a representative PI3K/Akt inhibitor. As shown in Figure 7B, co-treatment with LY294002 and DADS resulted in a marked increase in the accumulation of sub-G1 phase cells, indicating that the PI3K/Akt pathway plays a role in regulating DADS-induced apoptosis of DU145 cells.

### 2.8. Discussion

Although findings from recent studies have demonstrated that DADS, a main organosulfur component found in garlic, can suppress the growth of various cultured human cancer cell lines *in vitro*[15–22], the biochemical mechanisms by which this compound exerts its actions remain unclear. The present study aimed to identify the molecular signaling pathway of DADS involved in the induction of apoptosis in a human prostate cancer DU145 cell line. The results clearly demonstrated that DADS inhibits the growth of DU145 cells by induction of apoptotic cell death, which appears to account for its antiproliferating activity.

There are two classical pathways in apoptosis: an extrinsic pathway, which requires transmembrane death receptor-mediated interactions, and an intrinsic pathway, which initiates apoptosis *via* mitochondria-mediated stimuli [26–28]. Interaction between ligands and death receptors initiates the extrinsic pathway at the plasma membrane and, subsequently, the activation of caspase-8. Caspase-8 can directly activate downstream effector caspases such as caspase-3 and -7 [29–31]. In some cells, caspase-8 also mediates the intrinsic pathway via cleavage of Bid [32,33]. Changes in mitochondrial integrity by a broad range of physical and chemical stimuli can trigger the intrinsic pathway of apoptosis [23,24]. Mitochondrial dysfunction induces activation of caspase-9 and, subsequently, activates effector caspases, such as caspase-3. Following activation of caspase-3, cleavage of several specific substrates occurs, including PARP and β-catenin, which facilitates cellular disassembly and serves as a marker of cells undergoing apoptosis [34,35]. Our data indicated that DADS treatment resulted in the upregulation of the expression of FasL and DR4, crucial members of the extrinsic pathway. However, DADS inhibited levels of IAP family proteins such as XIAP and cIAP-1 (Figure 3), which reportedly block apoptosis due to direct inhibition by binding to and inhibiting several caspases [36].

Our results also revealed that DADS reduced the levels of procaspase-8 and -9, increased their catalytic activities, which involve the induction of initiator caspases of the extrinsic and intrinsic pathways, respectively (Figure 5). And induced the reduction of whole Bid proteins, which may relate to the activation of Bid (Figure 3). Furthermore, DADS markedly activated the key executioner, caspase-3 and the concomitant degradation of PARP and β-catenin, and DADS-induced apoptosis was associated with down-regulation of the antiapoptotic Bcl-2 protein, the loss of MMP and the translocation of cytochrome *c* from the mitochondria into the cytosol in the DU145 cells. Collectively, the present data suggest that both extrinsic and intrinsic pathways may have contributed, at least in part, to the DADS-induced apoptosis of the DU145 cells.

The MAPKs, a family of serine/threonine kinases, including ERK, JNK and p38 MAPK, play critical roles in cell survival and apoptosis in various cancer cells. It is well known that the activation of the p38 MAPK and JNK pathways leads to induction of apoptosis through the phosphorylation of a variety of proapoptotic downstream effectors, whereas the ERK pathway is more often associated with cell survival [37,38]. Moreover, the PI3K/Akt signal pathway plays critical roles in regulating cell survival and death in many physiological and pathological settings. The PI3K/Akt pathway is more often associated with cell survival through activation of antiapoptotic downstream effectors [39,40].

Our data indicated that phosphorylation of all three MAPKs increased rapidly in the DU145 cells in response to DADS and peaked after 1 to 3 h (Figure 6A). However, PD98059 and SB203580, specific inhibitors of ERK and p38 MAPK, respectively, did not inhibit DADS-induced apoptosis of the DU145 cells; while a potent inhibitor of JNK, SP600125, significantly inhibited DADS-induced apoptosis (Figure 6). The present results also demonstrated that DADS induces down-regulation of the PI3K/Akt signaling pathway, and that the inhibition of the PI3K/Akt pathway significantly increased DADS-induced apoptosis (Figure 7). Although more experiments are required to identify the full extent of the relationship between DADS and the cellular signaling pathways, the results indicate that DADS-induced apoptosis in DU145 cells is associated with the JNK and PI3K/Akt pathways.

## 3. Experimental Section

### 3.1. Reagents and Antibodies

DADS was purchased from LKT Laboratories (St. Paul, MN, USA), was dissolved in dimethyl sulfoxide (DMSO, Sigma-Aldrich, St. Louis, MO, USA) and adjusted to final concentrations using complete RPMI1640 (GIBCO-BRL, Gaithersburg, MD, USA). 3-(4,5-dimetylthiazol-2-yl)-2,5-diphenyl-tetrazolium (MTT), propidium iodide (PI), 5,5′, 6,6′-tetrachloro-1,1′,3,3′-tetraethyl-imidacarbocyanine iodide (JC-1) and DAPI were purchased from Sigma-Aldrich. Fetal bovine serum (FBS) and caspase activity assay kits were obtained from GIBCO-BRL and R&D Systems (Minneapolis, MN, USA), respectively. ERK-specific inhibitor, PD98059, JNK-specific inhibitor, SP600125, and p38 MAPK-specific inhibitor, SB203580, were purchased from Calbiochem (San Diego, CA, USA). The DNA staining kit (CycleTEST™ PLUS Kit) and enhanced chemiluminescence (ECL) kit were purchased from Becton Dickinson (San Jose, CA, USA) and Amersham (Arlington Heights, IL, USA), respectively. Antibodies specific for Fas, FasL, tumor necrosis factor-related apoptosis-inducing ligand (TRAIL), XIAP, cIAP-1, cIAP-2, Bcl-2, Bax, Bcl-xL, Bid, caspase-3, -8, -9, poly(ADP-ribose)polymerases (PARP) and β-catenin were obtained from Santa Cruz Biotechnology (Santa Cruz, CA, USA). Antibodies against for ERK, JNK, p38 MAPK, Akt, phosphor(p)-ERK, p-JNK, p-38MAPK and p-Akt were purchased from Cell Signaling (Beverly, MA, USA). Antibodies specific for DR 4 and DR5 were obtained from Calbiochem. Anti-actin antibody was obtained from Sigma-Aldrich.

### 3.2. Cell Culture and MTT Assay

The human prostate cancer line DU145 was purchased from the American Type Culture Collection (Rockville, MD) and maintained at 37 °C in humidified 95% air and 5% CO_2_ in RPMI1640 supplemented with 10% heat-inactivated FBS, 2 mM glutamine, 100 U/mL penicillin and 100 g/mL streptomycin. For the cell viability study, DU145 cells were grown to 70% confluence and treated with DADS. Control cells were supplemented with complete media containing 0.1% DMSO (vehicle control). Following treatment, cell viability was determined by use of the MTT assay, which is based on the conversion of MTT to MTT-formazan by mitochondrial enzymes. The effect of DADS on inhibition of cell growth was assessed as the percentage of cell viability, where vehicle-treated cells were considered 100% viable.

### 3.3. Nuclear Staining with DAPI

For DAPI staining, cells were washed with phosphate-buffered saline (PBS) and fixed with 3.7% paraformaldehyde in PBS for 10 min at room temperature. Fixed cells were washed with PBS and stained with a DAPI solution for 10 min at room temperature. Cells were then washed twice with PBS and analyzed using a fluorescence microscope (Carl Zeiss, Germany).

### 3.4. DNA Fragmentation Assay

In the DNA ladder assay, apoptotic DNA from cells treated with DADS for 48 h was selectively extracted using a DNA ladder assay kit (Suicide Track; Calbiochem). DNA was separated on a 1.0% agarose gel in TBE buffer (89 mM Tris-borate and 2 mM ethylene diamine tetraacetic acid (EDTA)) at 100 V. Ladders of DNA were visualized by staining with 0.1 μg/mL EtBr.

### 3.5. Flow Cytometric Analysis for Measurement of Sub-G1 Phase and MMP Values

For analysis of the cell cycle, cells were collected, washed with cold PBS and fixed in 75% ethanol at 4 °C for 30 min. The DNA content of the cells was measured using a DNA staining kit according to the manufacturer’s instructions. Then flow cytometric analyses were carried out using a flow cytometer (Becton Dickinson) and the relative DNA content determined using CellQuest software based on the presence of red fluorescence. The MMP was determined using the dual-emission potential-sensitive probe, JC-1. The cells were collected and incubated with 10 μM JC-1 for 20 min at 37 °C in the dark. The cells were then washed once with PBS and analyzed by a flow cytometer [41].

### 3.6. Protein Extraction and Western Blotting

Cells were harvested and washed twice in PBS at 4 °C. Total cells lysates were lysed in lysis buffer (40 mM Tris (pH 8.0), 120 mM, NaCl, 0.5% NP-40, 0.1 mM sodium orthovanadate, 2 μg/mL aprotinin, 2 μg/mL leupeptin and 100 μg/mL phenymethylsulfonyl fluoride). Supernatants were collected, and protein concentrations were then measured using protein assay reagents (Pierce, Rockford, IL, USA). Equal amounts of protein extracts were denatured by boiling at 95 °C for 5 min in sample buffer (0.5 M Tris-HCl, pH 6.8, 4% SDS, 20% glycerol, 0.1% bromophenol blue and 10% β-mercaptoethanol) at a ratio of 1:1, subjected to SDS-polyacrylamide gels and transferred to polyvinylidene difluoride membranes (Schleicher & Schuell, Keene, NH, USA) by electroblotting. Membranes were blocked with 5% non-fat dry milk in PBS with Tween 20 buffer (PBS-T) (20 mM Tris, 100 mM NaCl, pH 7.5 and 0.1% Tween 20) for 1 h at room temperature. Membranes were then incubated overnight at 4 °C with the primary antibodies, probed with enzyme-linked secondary antibodies and visualized using an ECL kit, according to the manufacturer’s instructions.

### 3.7. Caspase Activity Assay

Activities of caspases were determined by use of colorimetric assay kits, which utilize synthetic tetrapeptides (Asp-Glu-Val-Asp (DEAD) for caspase-3, Ile-Glu-Thr-Asp (IETD) for caspase-8, and Leu-Glu-His-Asp (LEHD) for caspase-9, respectively) labeled with p-nitroaniline (pNA). Briefly, DADS-treated and untreated cells were lysed in the supplied lysis buffer. Supernatants were collected and incubated with the supplied reaction buffer containing DTT and DEAD-pNA, IETD-pNA or LEHD-pNA as substrates at 37 °C. The reactions were measured by changes in absorbance at 405 nm using the VERSAmax tunable microplate reader [42].

### 3.8. Statistical Analysis

Unless otherwise indicated, each result is expressed as the mean ± SD of data obtained from triplicate experiments. Statistical analysis was performed using a paired Student *t*-test. Differences at *p* < 0.05 were considered statistically significant.

## 4. Conclusions

In summary, the present results demonstrated that DADS was capable of inhibiting cell proliferation and inducing apoptosis of DU145 cells through activation of the intrinsic caspase pathway, along with the death receptor-mediated extrinsic pathway. The apoptotic effects of DADS were also associated with the modulation of MAPKs and the PI3K/Akt signaling pathways. Taken together, these novel phenomena have not been previously described and provide important new insights into the possible biological effects of DADS.
